# Command vs. market in China’s energy intensity reduction strategies: Firm-level evidence

**DOI:** 10.1371/journal.pone.0263325

**Published:** 2022-02-10

**Authors:** Maoyong Fan, Jing Cao, Kuangyuan Zhang, Zhen Lei

**Affiliations:** 1 Department of Economics, Ball State University, Muncie, Indiana, United States of America; 2 Tsinghua University, School of Economics and Management, Tsinghua University, Beijing, China; 3 Santander Bank, Dorchester, Massachusetts, United States of America; 4 Department of Energy and Mineral Engineering and The EMS Energy Institute, Penn State University, University Park, Pennsylvania, United States of America; Center for International Climate and Environmental Research: CICERO, NORWAY

## Abstract

China has significantly reduced the energy consumption for per unit of GDP by using both command-and-controls or market-based strategies. This paper examines empirically the relative effectiveness and efficiency of command-and-control strategy (energy reduction target) vs. market-based strategy (electricity price). We find that (1) electricity price was similarly effective in reducing electricity intensity across firms, but government targets were more effective for firms that were more technologically outdated and energy intensive; and (2) government targets led to expenditures that were not useful in reducing energy intensity, suggesting inefficiency associated with targets. Despite the Chinese governments’ capacities and resources in directing and influencing enterprises, market-based approaches might still be more effective and efficient than command-and-control ones to reduce energy intensity.

## 1. Introduction

Pursuing sustainable development, and in particular addressing energy, environmental, water and climate challenges, require policy instruments that are either command-and-controls (which directly impose regulations) or market-based (which rely on price and market incentives). Market-based policies are often preferred by economists on the basis of efficiency, because they provide firms greater incentives and flexibility and enable maximization of net benefits [1]. However, few studies have examined the relative effectiveness of the two types of policies.

This paper is the first study to compare empirically the relative effectiveness and efficiency of command-and-control approaches versus market-based approaches in China’s effort to reduce its industrial energy intensity during the 11^th^ Five Year Plan period (2006–2010). It evaluates the relative effectiveness of government targets (a command-and-control approach) *versus* electricity price (a market-based instrument) in reducing industrial energy intensity in China. Energy intensity reduction is an important component in the endeavor to achieve zero net anthropogenic greenhouse gases (GHGs) emission by midcentury [2, 3]. Analyses that model pathways to net-zero emissions in 2050 in the U.S. conclude that in the next 10 years, the U.S. industrial sector must improve efficiency of energy and material use by 15 to 19 percent [4, 5].

China’s rapid economic growth has been largely driven by manufacturing industries that are often more energy intensive than their counterparts in advanced economies [6]. China surpassed the U.S. and became the world’s largest energy consumer in 2010, and its total energy consumption in 2018 was 46.4 billion tons of standard coal equivalents, approximately 23.6% of the world’s total primary energy consumption (See BP Statistical Review of World Energy 2019 and China Statistical Yearbook 2019). The rapid growth in energy consumption has given rise to severe environmental and health consequences, in addition to climate change, energy security and geopolitical concerns. China is the largest carbon emitter in the world in terms of total emissions. Approximately one million Chinese die from ambient air pollution according to the World Health Organization (WHO); and if air pollution were no more than the WHO recommended level, then it is estimated that 3.7 billion life-years would have been saved [7].

With a legacy of top-down planning, China’s approaches to tackling its energy, environmental and climate challenges have predominantly been target-based, manifested by numerous targets in China’s five-year plans and initiatives. Especially since the 11^th^ Five-Year Plan (FYP) covering 2006–2010, the central government has established national targets for pollution reduction and energy conservation [8]. These targets are set from top-down and reach to the lowest levels including townships and individual enterprises; and they are not pro forma goals − target fulfillments are important criteria in evaluating the performance of both local government officials and state-owned enterprise managers. These targets are implemented primarily with command-and-control policies, ranging from mandated technology upgrading to forced plant closures, though in many cases monetary incentives (e.g., subsidies and tax credits) are also involved.

Meanwhile, China has also experimented with market-based approaches to combatting energy and environmental issues [8]. The Differentiated Electricity Prices (DEP) policy, which sets different electricity prices for enterprises based on their sectors and energy intensity, has since 2004 been implemented to reduce firm energy intensity. Emission trading has been experimented with in selected cities and provinces, for SO_2_ since 2002 and for CO_2_ since 2012, respectively. More recently, China has converted the former pollution levy policy into an environmental tax system (though many argue that it is mainly fee-for-tax and not sufficient to curb pollution), and planned to launch a national carbon trading regime.

In this paper we examine an important question: how effective are China’s target-based approach *versus* price-based approach in addressing its sustainability challenges? We utilize a unique circumstance during the 11^th^ FYP period (2006–2010) when firms faced both government targets and price changes. More specifically in an effort to improve industrial energy saving and reduce emission, the Chinese government set a national target of 20% in energy intensity reduction (measured by energy consumption per 1,000-Yuan output) between 2006 and 2010. During the same period, electricity prices increased significantly, as the coal sector in China underwent deregulation that led to significant increase in coal prices. The increase in electricity price, interacting with the DEP policy, created variations in electricity prices not only for a given firm over time but also across firms in different sectors and locations.

We utilize a survey-based longitudinal firm level data and investigate relative effectiveness of the target-based approach (government targets on energy intensity reduction) *versus* the market-based instrument (electricity price increase), in reducing firm energy intensity. We also examine the impacts of government targets and electricity prices on firms’ self-reported expenditures (on energy saving, emission reduction, innovation, and technology adoption), to understand possible channels through which firms reduced energy intensity.

We find that both government targets and an increase in electricity price reduced firm electricity use intensity. However, an increase in electricity price reduced firm energy intensity across all types of firms, while government targets were more effective for firms that were less technologically advanced (and more energy intensive) than firms that were more technologically advanced. One possible explanation is that governments took a selective approach to implementing government targets by more closely monitoring firms that were more energy intensive. This suggests capacity and resource constraints in government implementation of targets [9, 10].

We also find that government targets, rather than electricity price increase, significantly enhanced firm investment and expenditures that were reportedly related to energy saving (including expenditures on process optimization, retrofitting old equipment, purchasing new equipment, labor costs and R&D). However, most of these expenditures were not associated with firm energy intensity reduction; and controlling for these expenditures, both government targets and electricity prices still significantly reduced firm energy intensity.

These results have two interesting implications. First, both prices and targets reduced firms’ energy intensity largely through other mechanisms than these expenditures, suggesting that there might be other “low hanging fruit” mechanisms, such as energy management [11], for firms to reduce energy intensity. This is consistent with another finding that firm profitability did not suffer during the period. Second, given that governments, when setting targets, often specify certain investments with financial incentives attached, firms might be incentivized to undertake these investments regardless of whether they are effective in reducing energy intensity, suggesting inefficiency associated with government targets. This is consistent with a recent qualitative interview-based study that concludes that “the reliance on binding environmental targets as the main domestic policy instrument in China has generated numerous undesirable consequences” [9].

To the best of our knowledge, our paper is the first to examine the relative effectiveness of the command-and-control approach *versus* market-based approach. Our analyses show that, even in China where governments possess nearly unparalleled capacity and authority in managing the economy and enterprises, market-based instruments might still work better than target-based approaches; government targets are likely to be implemented selectively among firms and lead to inefficient investments and expenditures. These findings offer important policy implications for energy, environmental and climate policies in China and in other countries as well (see the elaboration in the Policy Implications section).

It is noteworthy that we tested whether government targets and electricity prices were associated with certain firm characteristics, mostly finding no significant associations. This suggests that local governments impose targets and electricity prices on firms in their jurisdiction, in largely non-negotiable manner. Such non-negotiable imposition of government targets and electricity prices on firms, together with the panel data analysis that includes firm and province-by-year fixed effects, to large extent furnish a causal interpretation of the results. However, we still caution readers that our estimates could be biased due to omitted unobservables.

The remainder of the paper is organized as follows. In Section 2 we provide some background information about energy saving and emission reduction during China’s 11^th^ Five-Year Plan period. Section 3 describes the firm-level survey data. Section 4 presents and discusses the empirical results. Section 5 concludes.

## 2. Background

### 2.1 Targets for energy intensity reduction

China’s economic growth, largely driven by rapid industrialization, has been accompanied by expanding energy use, GHG emission and severe environmental degradation, which pose significant threats to human health, energy security and sustainable development [12]. China’s industrial sectors are responsible for the largest share of its end-use energy demand. During the 11^th^ FYP period (2006–2010), industrial energy use, mostly consisting of electricity generated in coal-fired power plants, increased from 1.6 billion tons of coal equivalent (TCE) to around 2.4 billion TCE [13]. According to the 2011 China Statistical Yearbook, in 2010 manufacturing energy use accounted for 73 percent of total energy consumption in China, more than three quarter of which was attributable to some energy intensive industries including iron and steel sector (29 percent), chemicals sector (17 percent), cement and other non-metallic minerals sector (15 percent), smelting and pressing of non-ferrous metals sector (8 percent), and petroleum refining sector (7 percent).

China made serious efforts to reduce manufacturing energy intensity during the 11^th^ FYP period. The central government set a national goal of 20 percent reduction in energy intensity by 2010, from the 2005 benchmark, and implemented it from top-down requiring provincial governments to set their annual targets accordingly [14]. To avoid shirking, at the beginning of a year, the governor of each province signed an individual responsibility contract with the central government, documenting specific energy intensity reduction targets for the province. The provincial government in turn determined targets for prefectural cities in the province, which then set targets for cities and counties in their respective jurisdictions [15, 16]. Whether a local government achieved its energy reduction target was taken into consideration in performance evaluation and promotion of government officials.

Enterprises, both state-owned enterprises (SOEs) and privately owned, were assigned annual energy intensity reduction targets. To achieve them, enterprises were required to establish an energy conservation department and an energy use reporting system, conduct energy audits, adopt incentives for energy conservation, provide training for employees, and upgrade or adopt technologies to reduce energy intensity. They were also required to report their energy consumption by fuel types to the National Bureau of Statistics (NBS), on a quarterly basis [17]. The targets for energy intensity reduction were primarily of a command-and-control approach, though governments also utilized financial instruments such as tax credits and subsidies to encourage enterprises to invest in energy intensity reduction.

### 2.2 Electricity price increase

In China the National Development and Reform Commission (NDRC) determines national baseline electricity prices. These are periodically revised to accommodate changes in prices of coal, a major source for electricity generation in China [18]. Based on national baseline prices, local governments set local baseline electricity prices in their jurisdiction. Local utility companies then use local baseline prices to set electricity prices for individual enterprises, which could differ across firms in the same locality (for example, the utility company could set a higher price for firms located in a newly established industrial park to which a new transmission line had to be built to deliver electricity).

During the 11^th^ FYP period, industrial electricity prices experienced significant increases, with the magnitude varying across locations and sectors, for two main reasons. First, during the 11^th^ FYP period, the coal sector underwent deregulation, leading to a sharp increase in coal prices (the national average coal price rose by 80 percent between 2007 and 2010). As a result, the electricity base prices had been on the rise during the period.

Second, as noted earlier, the DEP policy (implemented since 2003) set different electricity prices for firms based on their sectors and energy intensity, to curb growth in enterprises that were energy inefficient in sectors that were energy intensive. Firms in eight high energy consuming industries (electrolyzing aluminum, ferrous products, calcium carbide, caustic soda, cement, iron, and yellow phosphorus production and smelting) were classified into four categories: encouraged, permitted, restricted and eliminated. Firms in the first two categories received normal electricity prices, while firms in the latter two categories were imposed with higher (“differentiated”) prices. Local governments had some flexibility in determining the gap between normal and “differentiated” prices.

During the 11^th^ FYP, electricity price increase (due to coal price hikes) interacted with the DEP policy, creating significant variations in electricity prices over the years and among firms in different locations and sectors. Such variations in electricity prices allow us to estimate the impact of electricity prices on firm energy intensity.

## 3. Data

The data in our study was collected through a firm-level survey, conducted in 2010 with a representative sample of 1,000 firms in six provinces (Shandong, Shanxi, Jiangsu, Hebei, Jilin and Sichuan). The six provinces are in eastern, middle, and western China, respectively, reflecting the regional distribution in economic development across provinces. In each province, the survey questionnaire was sent to a sample of firms randomly drawn from the Industrial Enterprise Survey Database of the National Bureau of Statistics, which covered all manufacturing enterprises with an annual revenue of 5 million Yuan or above. This survey was conducted jointly by the Tsinghua Center for China in the World Economy (CCWE) and Chinese Academy of Social Sciences (CASS) in 2010, in collaboration with the provincial Development and Reform Commission (DRC) in the six provinces. The survey questionnaire was sent to the sampled firms from the provincial DRC office, and the raw response rate was nearly 100 percent.

**Fig 1:**
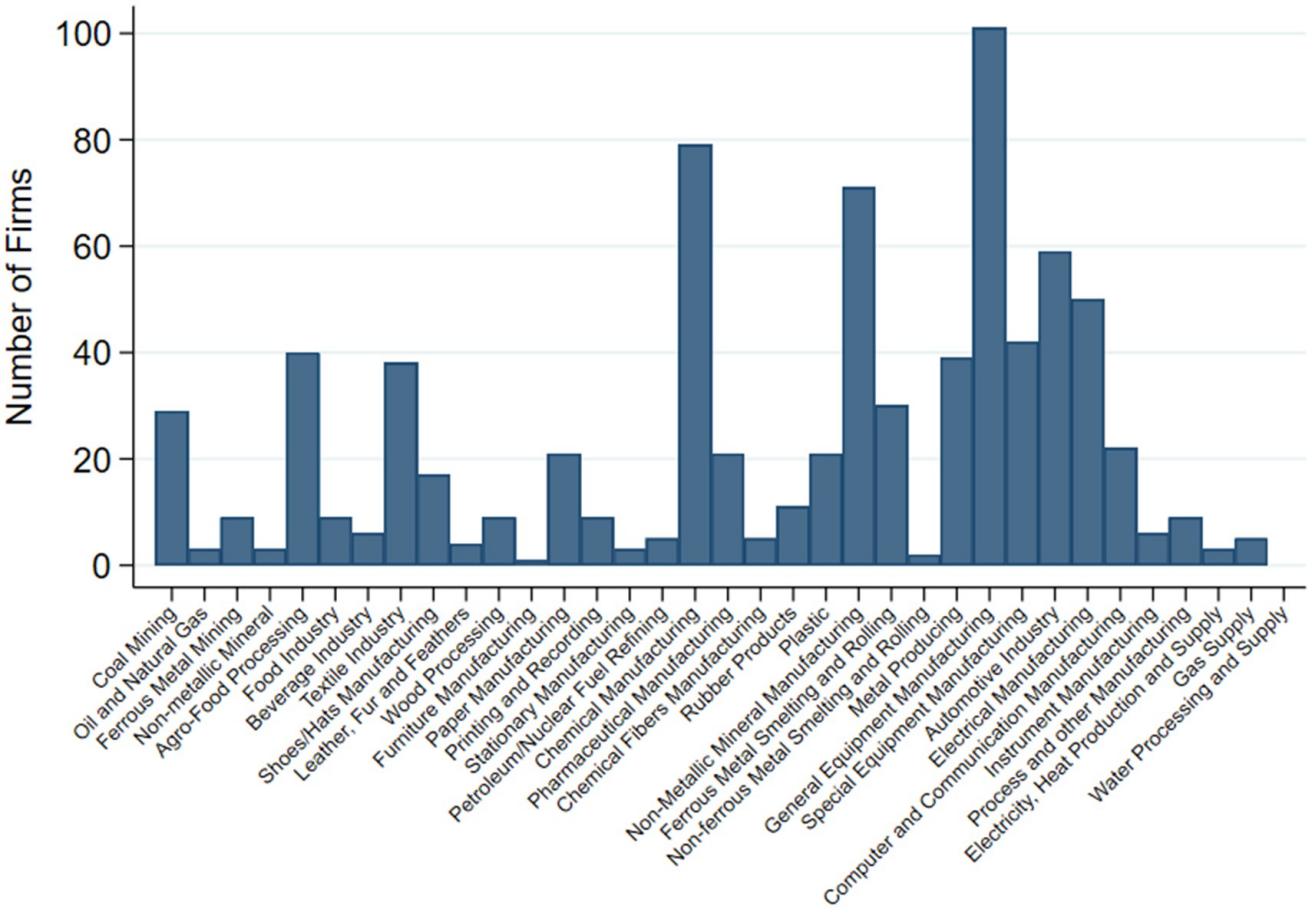
Firm distribution across industries.

After eliminating responses with incomplete data and responses by firms that had no significant electricity usage, the data include 782 firms that cover a wide range of industries including those in resource extraction, metal products, equipment, pulp and paper, chemicals, electronics, food and beverage, textiles, and other manufacturing activities. Fig 1 depicts the industry distribution of the firms in the data.

The survey questionnaire included qualitative questions about firm characteristics, as well as quantitative questions about government targets on energy intensity reduction. Energy consumption related questions include electricity usage and other forms of energy usage (such as coal, diesel and natural gas), prices of electricity, firm expenditures on energy saving and emission reduction, and expenditures on technology and innovation. Firms answered quantitative questions on an annual basis, for each year from 2005 to 2009. The data enable us to assemble a firm-level longitudinal/panel data, covering 782 firms through the years of 2006–2009 (during which period the firms were faced with government targets on energy intensity reduction), with reference to year 2005 as the base year.

## 4. Empirical results

### 4.1 Effectiveness of government targets and electricity prices on energy intensity

Electricity is the predominant form of energy used by the firms in the sample. Using electricity usage (kWh) per 1,000-Yuan output as the measurement of electricity intensity, we examined the effectiveness of government targets and electricity prices in reducing electricity intensity. Specifically, we run the following regression:

$$Y_{it}={\alpha T}_{it}+{\beta E}_{it}+{\delta P}_{it}{+\tau }_{i}+\pi_{pt}+\varepsilon_{it}$$ (1)

Where *Y*_*it*_ is electricity intensity for firm *i* in year *t*(*t* = 2006, 2007, 2008 and 2009). There are two key explanatory variables of interest: (1) *T*_*it*_, the *accumulative* target (in percentage points), relative to the base year 2005, on energy intensity reduction for firm *i* in year *t*; and (2) *E*_*it*_, the *relative* electricity price for firm *i* in year *t*, relative to the price in 2005. One concern is that government targets and electricity prices are highly correlated and the effects of the two policies are hard to separate. We examine the correlation between these two variables for each year and find that they are barely correlated, relieving us of the concern. Table A1 in the S1 Appendix shows that the correlation coefficients are less than 0.05 in absolute value for all years.

We controlled for *P*_*it*_, a vector of relative prices (relative to the prices in 2005) of both primary and secondary inputs and outputs, as these prices might impact firm energy intensity. We also included firm fixed effects (*τ*_*i*_) to control for unobserved firm characteristics that are time invariant, and province-by-year fixed effects (*π*_*pt*_) that account for policies in a province in a year that could impact firm energy intensity in that province in that year. *ε*_*it*_ is robust standard error and clustered at the firm level.

**Fig 2:**
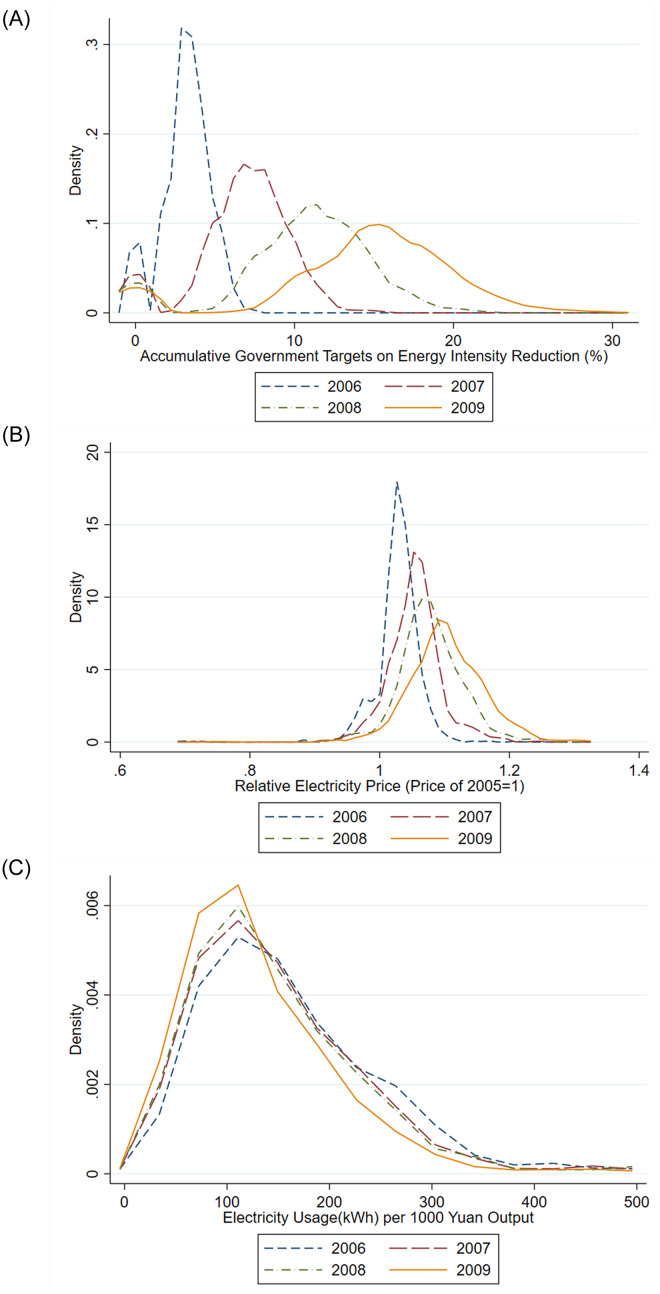
Government targets, electricity prices and electricity intensity, 2006–2009. **A**: Distributions of accumulative government targets (in percentage points), relative to 2005 (base year). **B**: Distributions of relative electricity prices, relative to 2005 (base year). **C**: Distributions of electricity intensity.

Fig 2 plots the distributions of accumulative government targets, relative electricity prices, and electricity use intensity, across firms and for each year during 2006–2009. Accumulative targets on energy intensity reduction (relative to the base year 2005) shifted to the right, and electricity prices (relative to the base year 2005) exhibited a clear pattern of increasing during the period, as shown in Fig 2A and 2B, respectively. The distribution of electricity intensity shifted leftward, suggesting that the firms became more energy efficient during the period (see Fig 2C). These figures suggest the effects of government targets and electricity price increases on electricity use intensity, which we next estimate in our econometric analyses. Also see Table A2 in the S1 Appendix for summary statistics of the variables in our analyses.

The econometric analysis results are presented in Table 1. Columns 1 and 2 show that government targets and electricity price increase significantly reduced firm electricity intensity. The results are robust whether or not we control for input and output prices. An increase of 0.01 (i.e., one percentage point) in government targets or relative electricity prices reduced firm electricy intensity by about 1 kwh per 1,000-Yuan output. This amounts to a reduction of 0.65%, given the average electricity intensity was 154 kwh per 1,000-Yuan output. Interestingly, the prices of inputs appear to have little impact on electricity intensity, but the price of the primary output is positively correlated with energy intensity.

**Table 1:** Effects of government targets and electricity prices.

	Electricity Use Intensity		Other Energy Use Intensity		Profitability	
	(1)	(2)	(3)	(4)	(5)	(6)
Government Target	-105.5***	-103.5***	-428.9***	-428.7***	90.2	100.5
	(40.0)	(39.5)	(163.9)	(164.7)	(91.3)	(90.7)
Electricity Price	-107.6***	-108.1***	39.2	39.5	-41.7	-44.3
	(29.5)	(29.6)	(71.8)	(71.9)	(47.2)	(47.1)
Price of Primary Input		-13.2		46.4**		54.8
		(10.7)		(20.7)		(37.3)
Price of Secondary Input		-5.2		43.0		-118.6
		(13.6)		(34.2)		(75.1)
Price of Primary Product		49.8**		-13.0		-24.4
		(22.0)		(57.6)		(50.2)
Price of Secondary Product		24.1		-28.2		208.7**
		(20.4)		(50.6)		(83.8)
Province-Year Fixed Effects	Yes	Yes	Yes	Yes	Yes	Yes
Firm Fixed Effects	Yes	Yes	Yes	Yes	Yes	Yes
Number of Firms	782	782	782	782	782	782
Observations	3,128	3,128	3,128	3,128	3,128	3,128
R-squared	0.18	0.18	0.03	0.03	0.02	0.02

*Notes*: Each column in the table represents a separate regression. The dependent variables are electricity intensity, electricity consumption (kWh) per 1,000-Yuan output, in Columns 1 and 2; other energy intensity, other energy consumption (kg oil equivalent) per 1,000-Yuan output, in Columns 3 and 4; and profitability, profit in Yuan per 1,000-Yuan output, in Columns 5 and 6. Government targets are accumulative targets (using 2005 as the base year), with value in decimals (i.e. a target of 10% takes 0.1 in value). Electricity prices are relative electricity prices (relative to the year 2005 price that is set as 1). Input and output prices are also relative prices to their respective prices in 2005. Heteroskedastic-consistent standard errors are clustered at the firm level and reported below the coefficients.

* significant at 10%,

** significant at 5%,

*** significant at 1%.

The sample firms also reported their annual use of other types of energy including coal, diesel and natural gas. In Columns 3 and 4 of Table 1 we examined how government targets and electricity prices affected intensity of other types of energy than electricity. We translated all other energy use into standard oil equivilient and calculated energy consumption per 1,000-Yuan output. The results show that government targets on energy intensity reduced firms’ intensity of other energy types, which makes sense as they were counted in government targets. In contrast, electricty prices had insignificant effects on the intensity of other types of energy, suggesting that electricity and other energy types are not good substitutes in production.

In Columns 4–5 of Table 1 we examined the impacts of government targets and electricty prices on firm profitability, measured by profit rate (profit per 1,000-Yuan output). The results suggest that government targets and electricty price hikes are not associated with firm profitability. The result suggests that there was ample room for firms to reduce electricity intensity, without hurting firm profits.

### 4.2 Heterogeneity in effectiveness of government targets and electricity prices

We next investigated whether government targets and electricity prices impacted firm electricity intensity differently across firms. We interacted *T*_*it*_ and *E*_*it*_ with indicators of firm characteristics in the regressions, as specified in Eq (2):

$$Y_{it}={\alpha T}_{it}+{\beta E}_{it}+{\theta I_{i}\times T}_{it}+{\rho I_{i}\times E}_{it}+{\delta P}_{it}{+\tau }_{i}+\pi_{pt}+\varepsilon_{it}$$ (2)

Where *I*_*i*_ is an indicator of firm characteristics.

Table 2 presents our results. We first distinguished firms that were not technological leaders, domestically or internationally, from those that were. The indicator of *Technology Status* equals to one for technological laggards (accounting for 36% of the sample firms) and zero otherwise. Firms in the laggard category were on average more electricity intensive than those technological leaders: 185.4 vs. 157.2 kWh per 1,000-Yuan output in 2005, which is a statistically significant difference (see Table A3 in the S1 Appendix). As shown in Column 1 of Table 2, the estimated coefficient for the interaction term, *Government Target × Technology Status*, is negative and statistically significant, whereas the coefficient for the interaction term, *Electricity Price × Technology Status*, is insignificant. This suggests that government targets reduced electricity intensity more significantly and in greater magnitude for firms that were not technological leaders, whereas increases in electricity price significantly reduced electricity intensity for both types of firms in similar ways. One possible explanation is that governments, when implementing targets, more closely monitored firms that were technological laggards and more energy intensive; consequently, targets were more effective in reducing energy intensity among technological laggards.

**Table 2:** Heterogeneous effects of government targets and electricity prices on electricity intensity.

	Technology Status	Newly Established	State Owned	Non-local Competitor
	(1)	(2)	(3)	(4)
Government Target	-54.1*	-100.2**	-100.9***	-99.0**
	(31.8)	(42.6)	(38.9)	(47.0)
Electricity Price	-96.7***	-126.5***	-106.7***	-64.9**
	(20.0)	(39.2)	(29.4)	(32.8)
Government Target × Indicator	-79.4**	-13.2	-34.0	-3.0
	(32.3)	(39.3)	(83.0)	(44.9)
Electricity Price × Indicator	-22.3	76.9	73.5	-55.1
	(47.9)	(48.9)	(56.6)	(54.0)
Input and Output Prices	Yes	Yes	Yes	Yes
Province-Year Fixed Effects	Yes	Yes	Yes	Yes
Firm Fixed Effects	Yes	Yes	Yes	Yes
Number of Firms	782	0.18	782	782
Observations	3,128	3,128	3,128	3,128
R-squared	0.19	0.19	0.18	0.18

*Notes*: Each column in the table represents a separate regression. The dependent variables are electricity consumption (kWh) per 1,000-Yuan output. Indicator in Column 1, *Technology Status*, equals 1 if the firm reports that it is not a technology leader. Indicator in Column 2, *Newly Established*, equals 1 if the firm was established after 2000. Indicator in Column 3, *State Owned*, equals 1 if the firm is a state-owned enterprise. Indicator in Column 4, *Non-local Competitor*, equals 1 if the firm’s major competitors are non-local (outside of the province where the firm is located). Government targets are accumulative targets using 2005 as the base year, with value in decimals (i.e. a target of 10% takes 0.1 in value). Electricity prices are relative prices to the year 2005 price that is set as 1. Input and output prices are also relative price to their respective prices in 2005. Heteroskedastic-consistent standard errors are clustered at the firm level and reported below the coefficients.

* significant at 10%,

** significant at 5%,

*** significant at 1%.

We then distinguished firms recently established from older firms, with the indicator of *Newly Established* being 1 for firms that were established after 2000 (constituting 34% of the sample firms). The two groups of firms were not significantly different in electricity intensity in 2005. As shown in Column 2 of Table 2, neither of the coefficients for the interaction terms Government Target * Newly Established and Electricity Price * Newly Established are significant. Thus, government targets and electricity prices appear to have similar effects on electricity intensity between new and older firms.

Next, we separated state-owned firms from other firms. The indicator of *State Owned* is one for state-owned firms (accounting for 6% of the firms in the sample). The results in Column 3 of Table 2 show that the effectiveness of both government targets and electricity prices was largely similar between state-owned firms and non-state firms; the coefficients for the interaction terms are both statistically insignificant. This is consistent with the notion that governments in China are powerful and thus government targets are effective for firms that governments supposedly have no direct controls. It is noteworthy that state-owned firms were similar to other firms in electricity intensity in 2005 (as shown in Table A3 in the S1 Appendix).

Finally, we distinguished firms reporting that their major competitors were not local (not in the same province) from those stating whose major competitors were in the same province, with the indicator of *Non-local Competitors* equal to one for the former group (constituting 66% of the sample firms). This is to test whether governments were more lenient in enforcing government targets for firms competing with non-local firms. The coefficients for the interaction terms are both statistically insignificant, indicating that government targets and electricity prices were effective in reducing electricity intensity for both types of firms in similar ways (Column 4 of Table 2). Again, no significant difference existed between the two groups of firms in electricity intensity in 2005.

Taken together, these results show that electricity prices were effective in reducing firm energy intensity in more homogenous ways than government targets. In particular, the effectiveness of government targets differs among firms with different technology status, but not along the other three dimensions of firm characteristics. This suggests that governments may face capacity and resource constraints and thus selectively enforce targets and more closely monitor firms that are more energy intensive [9, 10].

### 4.3 Channels in effectiveness of government targets and electricity prices

The survey asked firms to report their annual expenditures on process optimization, old equipment retrofitting, new equipment purchase and labor, specifically for energy saving energy and emission reduction. The firms also reported their expenditures on R&D and technology licensing/purchase, which were also related to energy intensity reduction.

To understand how government targets and electricity prices reduced energy intensity, we first examined how they impacted these expenditures, by regressing each expenditure on government targets and electricity prices:

$${ES}_{itk}={\alpha T}_{it}+{\beta E}_{it}+{\delta P}_{it}{+\tau }_{i}+\pi_{pt}+\varepsilon_{it}$$ (3)

Where *ES*_*itk*_ is expenditure of type *k* for firm *i* and year *t*, which was accumulative from year 2006 on, in accordance with both government targets and electricity prices being relative to the base year 2005,

Table 3 presents the results. Government targets significantly increased all four types of expenditures which were reportedly specific for saving energy and reducing emission, and also enhanced firm expenditure on R&D, but not technology licensing and purchase. In contrast, electricity prices did not have significant impacts on most expenditures, except that it reduced expenditure on process optimization, possibly because more optimized processes may use more electricity.

**Table 3:** Effect of government targets and electricity price on firm expenditures.

	Expenditure on Process Optimization	Expenditure on Old Equipment Retrofitting	Expenditure on New Equipment Purchase	Expenditure on Labor for Saving Energy	Expenditure on R&D	Expenditure on Technology Licensing/Purchase
	(1)	(2)	(3)	(4)	(5)	(6)
Government Target	25.5***	13.8**	46.3***	3.9***	99.0***	12.1
	(6.3)	(5.8)	(8.7)	(1.2)	(37.8)	(43.0)
Electricity Price	-9.5**	4.1	3.2	-0.8	31.0	27.2
	(4.5)	(3.8)	(6.2)	(0.7)	(18.9)	(22.9)
Input and Output Prices	Yes	Yes	Yes	Yes	Yes	Yes
Province-Year Fixed Effects	Yes	Yes	Yes	Yes	Yes	Yes
Firm Fixed Effects	Yes	Yes	Yes	Yes	Yes	Yes
Number of Firms	782	782	782	782	782	782
Observations	3,125	3,126	3,128	3,128	3,128	3,128
R-squared	0.77	0.71	0.65	0.81	0.21	0.15

*Notes*: Each column in the table represents a separate regression. The dependent variables are firm expenditures (Yuan): expenditures in Columns 1–4 are those firms reported specifically for saving energy and reducing emission, on process optimization (Column 1), retrofitting of old equipment (Column 2), new equipment purchase (Column 3), and labor used in energy saving (Column 4); expenditures in Columns 5–6 are on research and development (Column 5) and technology licensing and purchase (Column 6). Government targets are accumulative targets using 2005 as the base year, with value in decimals (i.e. a target of 10% takes 0.1 in value). Electricity prices are relative prices to the year 2005 price (which is 1). Input and output prices are also relative prices to their respective prices in 2005. Heteroskedastic-consistent standard errors are clustered at the firm level and reported below the coefficients.

* significant at 10%,

** significant at 5%,

*** significant at 1%.

Next, we explored whether these expenditures were associated with firms’ energy intensity and profitability. We run the following equation:

$$Y_{it}=\sum_{k=1}^{6}\gamma_{k}{ES}_{itk}+{\delta P}_{it}{+\tau }_{i}+\pi_{pt}+\varepsilon_{it}$$ (4)

Where *ES*_*itk*_ is the cumulative expenditure of type *k* in year *t* for firm *i*.

The results are presented in Table 4. Column 1 shows that, out of the six expenditures, only two expenditures, on retrofitting old equipment and purchasing new equipment, significantly reduced electricity intensity. The other four expenditures, of which three were positively correlated with government targets, had no significant effects on electricity intensity.

**Table 4:** Explore channels of effectiveness of government targets and electricity prices.

	Electricity Intensity		Other Energy Use Intensity		Profitability	
	(1)	(2)	(3)	(4)	(5)	(6)
Expenditure on Process Optimization	-25.4	22.0	72.8	538.6	1,100.3***	1,052.6**
	(212.8)	(204.7)	(673.0)	(829.3)	(422.8)	(430.2)
Expenditure on Old Equipment Retrofitting	-733.9***	-631.3**	-488.7	-51.8	1,300.3	1,277.9
	(265.4)	(257.8)	(448.5)	(559.7)	(961.9)	(969.0)
Expenditure on New Equipment Purchase	-319.5**	-235.3	-624.9	-174.9	799.7*	768.4*
	(150.2)	(145.2)	(411.3)	(583.8)	(426.1)	(419.0)
Expenditure on Labor for saving energy	-17.3	-4.5	45.6	83.8	9.7	9.2
	(59.3)	(58.0)	(87.6)	(100.7)	(87.4)	(86.1)
Expenditure on Research and Development	-23.5	-21.7	108.4*	104.6*	-18.8	-17.6
	(38.3)	(37.5)	(62.1)	(60.1)	(52.0)	(51.8)
Expenditure on Technology Licensing/Purchase	-644.7	-653.8	-1,502.7	-1,164.6	-2,526.9	-2,578.4
	(515.8)	(505.8)	(2,536.7)	(2,569.0)	(2,779.5)	(2,784.6)
Government Target		-82.0**		-432.6**		29.7
		(38.7)		(216.8)		(89.0)
Electricity Price		-99.2***		38.3		-43.8
		(26.0)		(68.8)		(46.7)
Input and Output Prices	Yes	Yes	Yes	Yes	Yes	Yes
Province-Year Fixed Effects	Yes	Yes	Yes	Yes	Yes	Yes
Firm Fixed Effects	Yes	Yes	Yes	Yes	Yes	Yes
Number of Firms	782	782	782	782	782	782
Observations	3,125	3,125	3,125	3,125	3,125	3,125
R-squared	0.18	0.19	0.02	0.03	0.03	0.03

*Notes*: The dependent variables are electricity intensity (in kWh per 1,000-Yuan output) in Columns 1 and 2, other energy intensity (in kg oil per 1,000-Yuan output) in Columns 3 and 4, and profitability (in profit per 1,000-Yuan output) in Columns 5 and 6. Government targets are accumulative targets using 2005 as the base year, with value in decimals (i.e. a target of 10% takes 0.1 in value). Electricity prices are relative prices to the year 2005 price that is set as 1. Input and output prices are also relative price to their respective prices in 2005. Heteroskedastic-consistent standard errors are clustered at the firm level and reported below the coefficients.

* significant at 10%,

** significant at 5%,

*** significant at 1%.

In Column 2, we add government targets (*T*_*it*_) and electricity prices (*E*_*it*_) as explanatory variables in the regression. The result shows that the coefficients for expenditures on retrofitting old equipment and purchasing new equipment became smaller in magnitude (and insignificant for the coefficient for expenditure on new equipment purchasing); meanwhile, the coefficient for government targets decreased by about one-fifth in magnitude but still remained significant, compared to the baseline results in Table 1. This suggests that on one hand government targets reduced electricity use intensity partly through incentivizing firms to retrofit old equipment and purchase new equipment, but on the other hand, there were perhaps other mechanisms through which government targets reduced firm electricity intensity.

The result, that government targets were positively associated with many expenditures that were reportedly specific for energy saving and emission reduction, but in practice did not reduce firm electricity intensity, is revealing. When Chinese governments impose targets on energy intensity reduction, they often specify, and even mandate, various measures (such as optimizing processes, retrofitting equipment, R&D, etc.) for firms to undertake, along with financial incentives such as subsidies and tax deduction. Our result shows that firms did respond to government mandates and incentives and undertake measures that were specified by the governments but in practice did not reduce firm energy intensity.

The coefficient of electricity prices in Column 2 of Table 4 is similar to the baseline result in Table 1, suggesting that electricity prices reduced firm electricity intensity through channels rather than these expenditures. This result is consistent with the finding in Table 3 that electricity price increases did not affect these expenditures.

Thus, electricity prices and government targets, by and large, reduced firm electricity intensity through other mechanisms than these expenditures. This suggests that there likely existed “low hanging fruits”—readily available and perhaps inexpensive measures, such as energy management, by which firms managed to reduce energy intensity. Considering this, there could be inefficiency associated with government targets, as targets increased firm expenditures that were reportedly specific for energy saving and emission reduction, but in practice not useful in reducing firm electricity intensity.

In Columns 3 and 4 of Table 4, we examined firms’ intensity of other types of energy. The results suggest that these expenditures had no significant impacts on other energy use intensity, except those expenditures on technology licensing and purchase were positively associated with other energy intensity (significantly at the 10% level). The coefficient of government target here is similar to the baseline result in Table 1, implying that government targets reduced other energy intensity through channels other than these expenditures.

Finally, in Columns 5 and 6 of Table 4, we examined firm profitability. The results show that expenditures on process optimization and new equipment purchase increased firm profitability. This suggests that firms may have taken advantage of financial incentives associated with government targets and undertaken process optimization and new equipment purchase to increase profits, in the name of energy and pollution reduction.

### 4.4 Potential bias in estimation

In the analysis, the panel data structure allows us to use firm fixed effects and province-year fixed effects to control for the omitted variable bias to some extent. The firm fixed effects control for time invariant unobserved firm characteristics that are related to firm energy intensity. The province-year fixed effects control for provincial energy and environmental policies (e.g., province leaders’ enforcement of environmental policies) that could affect the energy intensity of all firms in the province. We also tested whether government targets and electricity prices were associated with certain firm characteristics, mostly finding no significant associations (see Table A4 in the S1 Appendix). This is consistent with the notion that Chinese governments play dominant roles and usually impose targets and electricity prices on firms in a largely non-negotiable manner. Such non-negotiable imposition of government targets and electricity prices on firms, together with including firm and province-by-year fixed effects in the panel data analysis, to a substantial extent alleviates concerns about estimation bias.

However, there could be unobserved factors that lead to estimation bias. For example, a local government that focuses on economic growth rather than environmental protection could impose more lenient targets and/or lower electricity prices on firms in its jurisdiction. We caution readers that the estimates in the paper might be biased due to omitted unobservables.

## 5. Conclusion

This paper compares the relative effectiveness of government targets (a command-and-control instrument) *versus* electricity prices (a market-based approach) in reducing firm energy intensity in China during the 11^th^ FYP period (2006–2010). There are three interesting findings. First, an increase in electricity price was similarly effective in reducing electricity intensity across firms, but government targets were more effective for firms that were less technologically advanced and more energy intensive. Second, government targets led to firm investments and expenditures that, though presumably related to energy intensity reduction and thus mandated and/or incentivized by governments, were not practically effective in reducing energy intensity. This suggests inefficiencies associated with government targets. Third, electricity prices and government targets seemed to reduce firm electricity intensity through readily available measures of “low hanging fruits.”

## 6. Policy implications

Our study shows that, despite the Chinese governments’ capacity and resources in managing and directing economic activities, target-based approaches may still be less effective and efficient than market-based instruments. This insight is important for energy, environmental and climate policies in China and in other countries, even today when an energy transition from fossil fuel to renewable energy has been underway [3, 19] and the COVID-19 pandemic might have caused lasting impacts on energy consumption [20].

First, China’s industrial energy intensity reduction during the 11^th^ FYP period has been widely viewed as a prominent success: energy efficiency was improved by 19.1% nationwide during the 11^th^ FYP period in China, which was achieved despite a rebound in energy use and carbon emissions in later years of the 11th FYP period due to the stimulus in response to the 2008 Great Recession. Our study sheds empirical light and insights on which mechanisms were more effective and efficient in achieving this success. This is important and timely for China’s energy, environmental and climate policies and in particular its clean energy transition strategies to achieve the intended nationally determined contributions (INDC) in carbon emission reduction. China, with its legacy of government planning and micro-management, has a greater tendency to employ target-based policies. In recent years, for instance, to combat air pollution the Chinese government has announced such policies as the “2+26” and “Atmospheric Ten” policies that set targets on air pollution reduction, while market-based instruments have been less emphasized. Our results, however, put some cautionary notes on China’s inclination to rely heavily on targets and command-and-control policies in achieving its sustainable development goals.

Second, our analysis on the effectiveness of command-and-control *versus* market-based approaches in China is informative for other countries’ policies to reduce energy intensity, and their energy, environmental and climate policies in general. Developed countries and the majority of developing countries have taken measures toward decreasing energy intensity, as it has multiple benefits including improving energy security, economic competitiveness, air quality and public health, and lowering greenhouse gas emissions [21]. While policy options should be country specific and essentially an empirical matter, and clearly there are no one-size-fits-all answers, our findings are informative for other developing countries where governments are unlikely to possess as great state capacity and resources as the Chinese government does [22].

It is noteworthy that our paper focuses government policies, separating command-and-controls from prices, but firms might have other incentives and reasons, such as corporate social responsibility [23], to improve energy and environmental performance. It is important to take those incentives into account when designing policies addressing energy and environmental and climate challenges.

## Supporting information

S1 File (RAR)

S1 Appendix (DOCX)

## References

[1] Hahn Robert W. and Stavins Robert N. "Economic Incentives for Environmental Protection: Integrating Theory and Practice." *The American Economic Review*, 1992, 82(2), pp. 464–68.

[2] Allen, Myles R; Babiker, Mustafa; Chen, Yang; de Coninck, Heleen; Connors, Sarah; van Diemen, Renée; et al. "Global Warming of 1.5°C: An IPCC Special Report on the Impacts of Global Warming of 1.5°C above Pre-Industrial Levels and Related Global Greenhouse Gas Emission Pathways" IPCC, 2018.

[3] National Academies of Sciences, Engineering and Medicine. "Accelerating Decarbonization of the Us Energy System," The National Academies Press: Washington, DC, USA, 2021.

[4] Larson, E; Greig, C; Jenkins, J; Mayfield, E; Pascale, A; Zhang, C; et al. "Net-Zero America by 2050: Potential Pathways, Deployments and Impacts (No. Interim Project Report)." *Princeton University*, *Princeton*, *NJ*, *USA*, 2020.

[5] Nadel, Steven and Ungar, Lowell. "Halfway There: Energy Efficiency Can Cut Energy Use and Greenhouse Gas Emissions in Half by 2050." *Report u1907 american council for an energy-efficient economy*, 2019.

[6] Hsieh Chang-Tai and Klenow Peter J. "Misallocation and Manufacturing Tfp in China and India*." *The Quarterly Journal of Economics*, 2009, 124(4), pp. 1403–48.

[7] Ebenstein Avraham; Fan Maoyong; Greenstone Michael; He Guojun and Zhou Maigeng. "New Evidence on the Impact of Sustained Exposure to Air Pollution on Life Expectancy from China’s Huai River Policy." *Proceedings of the National Academy of Sciences*, 2017, 114(39), pp. 10384–89. doi: 10.1073/pnas.1616784114 28893980 PMC5625887

[8] Schreifels Jeremy J; Fu Yale and Wilson Elizabeth J. "Sulfur Dioxide Control in China: Policy Evolution During the 10th and 11th Five-Year Plans and Lessons for the Future." *Energy Policy*, 2012, 48, pp. 779–89.

[9] Kostka Genia. "Command without Control: The Case of China’s Environmental Target System." *Regulation & Governance*, 2016, 10(1), pp. 58–74.

[10] Wang Alex L. "The Search for Sustainable Legitimacy: Environmental Law and Bureaucracy in China." *Harv*. *Envtl*. *L*. *Rev*., 2013, 37, pp. 365.

[11] Cooremans Catherine and Schönenberger Alain. "Energy Management: A Key Driver of Energy-Efficiency Investment?" *Journal of Cleaner Production*, 2019, 230, pp. 264–75.

[12] Ebenstein Avraham; Fan Maoyong; Greenstone Michael; He Guojun; Yin Peng and Zhou Maigeng. "Growth, Pollution, and Life Expectancy: China from 1991–2012." *American Economic Review*, 2015, 105(5), pp. 226–31.

[13] The statistics are from the China State Council’s Twelfth Five-Year Plan. http://www.miit.gov.cn/n11293472/n11293877/n13434815/n13434832/n14476034.files/n14474195.pdf.

[14] Hu Yuan. "Implementation of Voluntary Agreements for Energy Efficiency in China." *Energy Policy*, 2007, 35 *(*2007*)*, pp. 5541–48.

[15] Cao Jing and Karplus Valerie J. "Firm-Level Determinants of Energy and Carbon Intensity in China." *Energy Policy*, 2014, 75, pp. 167–78.

[16] Price Lynn; Wang Xuejun and Yun Jiang. "The Challenge of Reducing Energy Consumption of the Top-1000 Largest Industrial Enterprises in China." *Energy Policy*, 2010, 38(11), pp. 6485–98.

[17] National Development and Reform Commission (NDRC). "Notice of Issuance of the Thousand Enterprise Energy Saving Action Implementation Plan," National Development and Reform Commission, 2006.

[18] Liu Ming-Hua; Margaritis Dimitris and Zhang Yang. "Market-Driven Coal Prices and State-Administered Electricity Prices in China." *Energy Economics*, 2013, 40, pp. 167–75.

[19] Abbasi Kashif Raza; Adedoyin Festus Fatai; Abbas Jaffar and Hussain Khadim. "The Impact of Energy Depletion and Renewable Energy on CO2 Emissions in Thailand: Fresh Evidence from the Novel Dynamic ARDL Simulation." *Renewable Energy*, 2021, 180, pp. 1439–50.

[20] Jiang Peng; Van Fan Yee; Klemeš Jiří Jaromír. "Impacts of COVID-19 on energy demand and consumption: Challenges, lessons and emerging opportunities." *Applied Energy*, 2021, 285, 116441. doi: 10.1016/j.apenergy.2021.116441 33519038 PMC7834155

[21] Azhgaliyeva Dina; Liu Yang and Liddle Brantley, "An Empirical Analysis of Energy Intensity and the Role of Policy Instruments." *Energy Policy*, 2020, 145, 111773.

[22] Blackman Allen and Harrington Winston. "The Use of Economic Incentives in Developing Countries: Lessons from International Experience with Industrial Air Pollution." *The Journal of Environment & Development*, 2000, 9(1), pp. 5–44.

[23] Abbas Jaffar, et al. “The Effects of Corporate Social Responsibility Practices and Environmental Factors through a Moderating Role of Social Media Marketing on Sustainable Performance of Business Firms”. *Sustainability*, 2019, 11(12), 3434.

