# Supplementary material for: Command vs. market in China’s energy intensity reduction strategies: Firm-level evidence
Source: PLoS One. 2022 Feb 10;17(2):e0263325. doi: 10.1371/journal.pone.0263325 (PMC8830638; doi:10.1371/journal.pone.0263325)
Supplement: S1 Appendix — (DOCX) [file pone.0263325.s002.docx]

**Appendix**

**Command vs. Market in China’s Energy Intensity Reduction Strategies:**

**Firm-level Evidence**

**Table A1. Correlation between energy reduction targets and electricity prices**

| Year | Correlation Coefficients |
| --- | --- |
| 2006 | -0.0128 |
| 2007 | -0.0453 |
| 2008 | -0.0314 |
| 2009 | -0.0322 |

**Table A2: Summary Statistics**

| N=3,128, Number of Firm=782 | Mean | S.D. | P25 | P75 |
| --- | --- | --- | --- | --- |
|  |  |  |  |  |
| Electricity Consumption (kWh) Per 1,000 Yuan Output | 154 | 119 | 87 | 191 |
| Other Energy Consumption (kg oil equivalent) Per 1,000 Yuan Output | 96 | 539 | 0 | 6 |
| Profit (Yuan) Per 1,000 Yuan Output | 52 | 90 | 8 | 83 |
| Annual Government Target (%) on Energy Intensity Reduction, Relative to the Previous Year | 3.49 | 1.56 | 3.00 | 4.40 |
|  |  |  |  |  |
| Accumulative Government Target (%) on Energy Intensity Reduction, Relative to Year 2005 | 8.72 | 5.77 | 4.00 | 13.00 |
| Electricity Price (Yuan/kwh) | 0.79 | 0.10 | 0.72 | 0.86 |
| Relative Electricity Price (Year 2005=1) | 1.07 | 0.05 | 1.03 | 1.09 |
| Relative Price of Major Input (Year 2005=1) | 1.08 | 0.10 | 1.03 | 1.11 |
| Relative Price of Secondary Input (Year 2005=1) | 1.07 | 0.09 | 1.03 | 1.10 |
| Relative Price of Major Product (Year 2005=1) | 1.06 | 0.06 | 1.03 | 1.08 |
| Relative Price of Secondary Product (Year 2005=1) | 1.05 | 0.07 | 1.00 | 1.08 |
| Expenditure on Research and Development (1,000 Yuan) | 6,144 | 55,454 | 0 | 724 |
| Expenditure on Technology Licensing/Purchase (1,000 Yuan) | 1,424 | 6,612 | 0 | 0 |
| Expenditures on Energy Saving and Emission Reduction (1,000 Yuan) | 7,323 | 57,664 | 400 | 2,655 |
| Process Optimization (1,000 Yuan)^ψ^ | 1,816 | 14,124 | 94 | 838 |
| Old Equipment Retrofitting (1,000 Yuan)^ψ^ | 1,112 | 6,569 | 78 | 546 |
| Labor Cost (1,000 Yuan) | 355 | 2,704 | 19 | 156 |
| New Equipment Purchase (1,000 Yuan) | 4,042 | 47,159 | 6 | 978 |
|  |  |  |  |  |
| *Notes:* In the final sample, there are 782 independent firms and 3,128 firm-year observations. The money value is in 2005 Yuan for all price and expenditure variables.  ^ψ^ The number of observations for process optimization and old equipment retrofitting is 3,125 and 3,126 respectively. | | | | |

**Table A3: Electricity use intensity comparisons**

Table A3 explores whether there are significant differences in electricity intensity between firms of different characteristics, for year 2005 (prior to the 11^th^ FYP). The indicator of *Technology Status* distinguishes firms that were not technological leaders, domestically or internationally, from those that were, being 1 for technological laggards and zero otherwise. The results show that firms that were less technology advanced were significantly more energy intensive than firms that were technology leaders (Column 1), but there was no significant difference in energy intensity between firms along other firm characteristics (Columns 2-4).

Table A3: Electricity use intensity comparisons

|  | Technology Status | Newly Established | State  Owned | Non-local Competitor |
| --- | --- | --- | --- | --- |
|  | (1) | (2) | (3) | (4) |
| **Electricity Intensity** |  |  |  |  |
| Mean (Indicator=0) | 157.2 | 176.5 | 175.4 | 168.7 |
| Mean (Indicator=1) | 185.4 | 154.6 | 175.2 | 178.7 |
| T-statistics of mean difference | -2.6 | 1.0 | 0.0 | -0.9 |
| P-value | 0.0 | 0.3 | 1.0 | 0.4 |
|  |  |  |  |  |

Notes: Indicator *Technology Status* in Column 1 equals 1 if the firm reports that it is not a technology leader. Indicator *Newly Established* in Column 2 equals 1 if the firm was established after 2000 (in 2001 or after). Indicator *State Owned* in Column 3 equals 1 if the firm is a state-owned enterprise. Indicator *Non-local Competitor* in Column 4 equals 1 if the firm’s major competitors are non-local (outside of the province where the firm is located).

**Table A4. Associations between firm characteristics and targets and electricity prices**

In Table A4 we tested whether government target and electricity price for a firm in year *t* (t=2006, 2007, 2008, 2009, separately) were associated with the four firm characteristics (Technology Status, Newly Established, State Owned and Non-local competitors) and its electricity intensity in the previous year *t-1*. The indicator of *Technology Status* distinguishes firms that were not technological leaders, domestically or internationally, from those that were, being 1 for technological laggards and zero otherwise. The indicator of *Newly Established* distinguishes firms recently established from older firms, being 1 for firms that were established after 2000. The indicator of *State Owned* separates state-owned firms from other firms, being 1 for state-owned firms. The indicator of *Non-local Competitors* separates firms reporting that their major competitors were not local (not in the same province) from those stating whose major competitors were in the same province, equal to 1 for the former group.

We find no such significant associations that are consistent in all four years, except that there is a significant and consistent positive correlation between government target in year *t* and electricity intensity in year *t-1,* which nevertheless is not economically meaningful (the mean electricity intensity in the data is 154 kwh per 1000 Yuan output; and based on the estimate, even a firm that is 100 kwh per 1000 Yuan output more intensive, its annual target will be increased by governments by 0.1%, which is very small compared to the average annual target of 8.72%). The association between firm ownership and electricity prices is also significantly positive in three of the four year, but again the difference is not meaningful (the difference is 0.0089 given the average relative electricity price is 1.07 in the data). The lacking in significant and meaningful association between firm characteristics and government targets and electricity prices is consistent with the notion that Chinese governments play dominant roles and impose targets and prices on firms.

Table A4. Associations between firm characteristics and targets and electricity prices

|  | Annual Target | | | | Relative Electricity Price | | | |
| --- | --- | --- | --- | --- | --- | --- | --- | --- |
|  | 2006 | 2007 | 2008 | 2009 | 2006 | 2007 | 2008 | 2009 |
|  | (1) | (2) | (3) | (4) | (5) | (6) | (7) | (8) |
|  |  |  |  |  |  |  |  |  |
| Technology Status | 0.05 | -0.06 | -0.03 | -0.04 | -0.01 | -0.22 | -0.38 | -1.02** |
|  | (0.11) | (0.11) | (0.12) | (0.12) | (0.25) | (0.32) | (0.37) | (0.43) |
| Newly Established | 0.04 | 0.03 | 0.07 | 0.13 | -0.05 | -0.50 | -0.13 | 0.03 |
|  | (0.11) | (0.12) | (0.12) | (0.12) | (0.26) | (0.33) | (0.38) | (0.44) |
| State Owned | 0.33 | 0.28 | 0.18 | 0.24 | 0.89* | 1.45** | 1.36* | 0.94 |
|  | (0.22) | (0.24) | (0.24) | (0.25) | (0.51) | (0.66) | (0.78) | (0.90) |
| Non-local Competitor | -0.04 | 0.04 | 0.04 | 0.10 | 0.22 | 0.10 | 0.26 | 0.15 |
|  | (0.11) | (0.12) | (0.12) | (0.12) | (0.25) | (0.32) | (0.38) | (0.44) |
| Electricity Intensity_t-1_ | 0.0011*** | 0.0013*** | 0.0019*** | 0.0019*** | 0.0006 | 0.0010 | 0.0019 | 0.0010 |
|  | (0.0003) | (0.0004) | (0.0005) | (0.0005) | (0.0008) | (0.0010) | (0.0015) | (0.0019) |
|  |  |  |  |  |  |  |  |  |
| Observations | 782 | 782 | 782 | 782 | 782 | 782 | 782 | 782 |
| R-squared | 0.07 | 0.09 | 0.09 | 0.08 | 0.01 | 0.02 | 0.02 | 0.02 |
| *Notes:* The dependent variables are annual targets and relative electricity prices (relative to the year 2005 price). In the regressions, to make coefficient magnitude to be larger, the values of both dependent variables are the original value multiplied by 100 (i.e. a target of 10% taking the value of 10 and a relative electricity price of 1.10 taking the value of 110). The independent variables include lagged electricity intensity and firm characteristics: Technology Status (equal to 1 if the firm reports that it is not a technology leader), Newly Established (equal to 1 if the firm was established after 2000), State Owned (equal to 1 if the firm is a state-owned enterprise), and Non-local Competitor (equal to 1 if the firm’s major competitors are non-local, outside of the province where the firm is located). Six province dummies are included. * significant at 10%, ** significant at 5%, *** significant at 1%. | | | | | | | | |
